# Beamformer Source Analysis and Connectivity on Concurrent EEG and MEG Data during Voluntary Movements

**DOI:** 10.1371/journal.pone.0091441

**Published:** 2014-03-11

**Authors:** Muthuraman Muthuraman, Helge Hellriegel, Nienke Hoogenboom, Abdul Rauf Anwar, Kidist Gebremariam Mideksa, Holger Krause, Alfons Schnitzler, Günther Deuschl, Jan Raethjen

**Affiliations:** 1 Department of Neurology, Christian-Albrechts-University, Kiel, Germany; 2 Institute for Circuit and System Theory, Christian-Albrechts-University, Kiel, Germany; 3 Department of Neurology, Heinrich-Heine University, Dusseldorf, Germany; University of Rome, Italy

## Abstract

Electroencephalography (EEG) and magnetoencephalography (MEG) are the two modalities for measuring neuronal dynamics at a millisecond temporal resolution. Different source analysis methods, to locate the dipoles in the brain from which these dynamics originate, have been readily applied to both modalities alone. However, direct comparisons and possible advantages of combining both modalities have rarely been assessed during voluntary movements using coherent source analysis. In the present study, the cortical and sub-cortical network of coherent sources at the finger tapping task frequency (2–4 Hz) and the modes of interaction within this network were analysed in 15 healthy subjects using a beamformer approach called the dynamic imaging of coherent sources (DICS) with subsequent source signal reconstruction and renormalized partial directed coherence analysis (RPDC). MEG and EEG data were recorded simultaneously allowing the comparison of each of the modalities separately to that of the combined approach. We found the identified network of coherent sources for the finger tapping task as described in earlier studies when using only the MEG or combined MEG+EEG whereas the EEG data alone failed to detect single sub-cortical sources. The signal-to-noise ratio (SNR) level of the coherent rhythmic activity at the tapping frequency in MEG and combined MEG+EEG data was significantly higher than EEG alone. The functional connectivity analysis revealed that the combined approach had more active connections compared to either of the modalities during the finger tapping (FT) task. These results indicate that MEG is superior in the detection of deep coherent sources and that the SNR seems to be more vital than the sensitivity to theoretical dipole orientation and the volume conduction effect in the case of EEG.

## Introduction

EEG and MEG are two non-invasive techniques with a high temporal resolution for imaging the neuronal activity in the brain. The integration of both these modalities have been shown to be more advantageous than using them separately in previous studies [1]–[10]. The differences in SNR and sensitivity for MEG and/or EEG have been examined [11]–[14] either in pure simulations or simulated data from real recordings. Especially in MEG, the sensitivity is different for systems with only magnetometers or gradiometers (planar or axial) [15], [16]. It is well established that MEG recordings yield higher signal-to-noise ratios than EEG recordings whereas its sensitivity to more radially oriented dipoles is minimal which could be a disadvantage especially for detecting deep sub-cortical sources [17], [18]. However, in coherent source analysis approaches it has been clearly shown that MEG is able to detect oscillatory network components even in the thalamic region [19], [20]. In previous studies direct comparisons between MEG, EEG and the combination of the two are lacking for such coherent source analysis. Thus, it is not clear if the better SNR of MEG data improves the lack of sensitivity to the more radially oriented dipoles in deep brain structures. We chose a voluntary task like the finger tapping task, a well-defined task in which the central networks that are involved are well described [21]–[24]. This allowed us to assess the quality of the source analyses performed on MEG, EEG and the combination of the two modalities. In order to detect the oscillatory central networks involved in this task, we computed coherence between simultaneously recorded 128-channel EEG with 306 MEG and forearm electromyography (EMG) and performed coherent source analysis using DICS [19]. In the next step, we analysed the direction of information flow between the source signals using the RPDC [25]. Both methods are well established and have been extensively applied on EEG and MEG data [19], [20], [24]–[28].

## Subjects and Methods

### 2.1 Subjects

Eight male and seven female healthy volunteers participated in this study. All gave written informed consent. The study was approved by the Ethics Committee, Medical Faculty, University of Kiel. Age ranged from 23 to 39 yr. (mean: 29.81±5.25). All were right handed. Subjects were seated in a comfortable chair in a slightly reclined position. Both forearms were supported by firm armrests up to the wrist joints. The hands were held outstretched against gravity, and the subjects were asked to keep their eyes open and fixed on a point about 2 m away.

Muscle activity was recorded by surface electromyography from the right hand forearm flexors and extensors using silver chloride electrodes. MEG and EEG were recorded simultaneously using an Elekta Neuromag system. The EEG data was recorded with 128 electrodes, the MEG data from 306 sensors containing a triple sensor array, which optimally combines the focal sensitivity of 204 planar gradiometers and the widespread sensitivity of 102 magnetometers. Data were stored in a computer and analyzed off-line. Individual recordings were of 4 to 5 minutes duration. The subjects were asked to perform a rhythmic right index finger tapping movement in a self-paced manner. The rhythmic movements were checked for each subject by looking at the EMG activity online to have at least 2–4 bursts per second.

### 2.2 Data Pre-processing

The simultaneous recording of MEG, EEG and EMG were sampled at 1000 Hz and band-pass filtered (EMG 30–200 Hz; MEG and EEG 0.05–200 Hz). EMG was full-wave rectified; the combination of band-pass filtering and rectification is the common demodulation procedure for tremor EMG [29]. Due to some recent differences in opinion about the rectification of the EMG signals as mentioned in [30]–[32], we estimated the EMG power spectrum with and without rectification and also cortico-muscular coherence with a single EEG/MEG channel on the contralateral motor cortex (e.g., C3/MEG 0231). Each record was segmented into a number of 1 s - long high-quality epochs (L) discarding all those data sections with visible artifacts. For each task, depending on the length (N) of the recording and the quality of the data, between 250 to 260 1-s segments (M) were used for analysis such that N = LM.

### 2.3 Realistic Head Models

**Figure 1:**
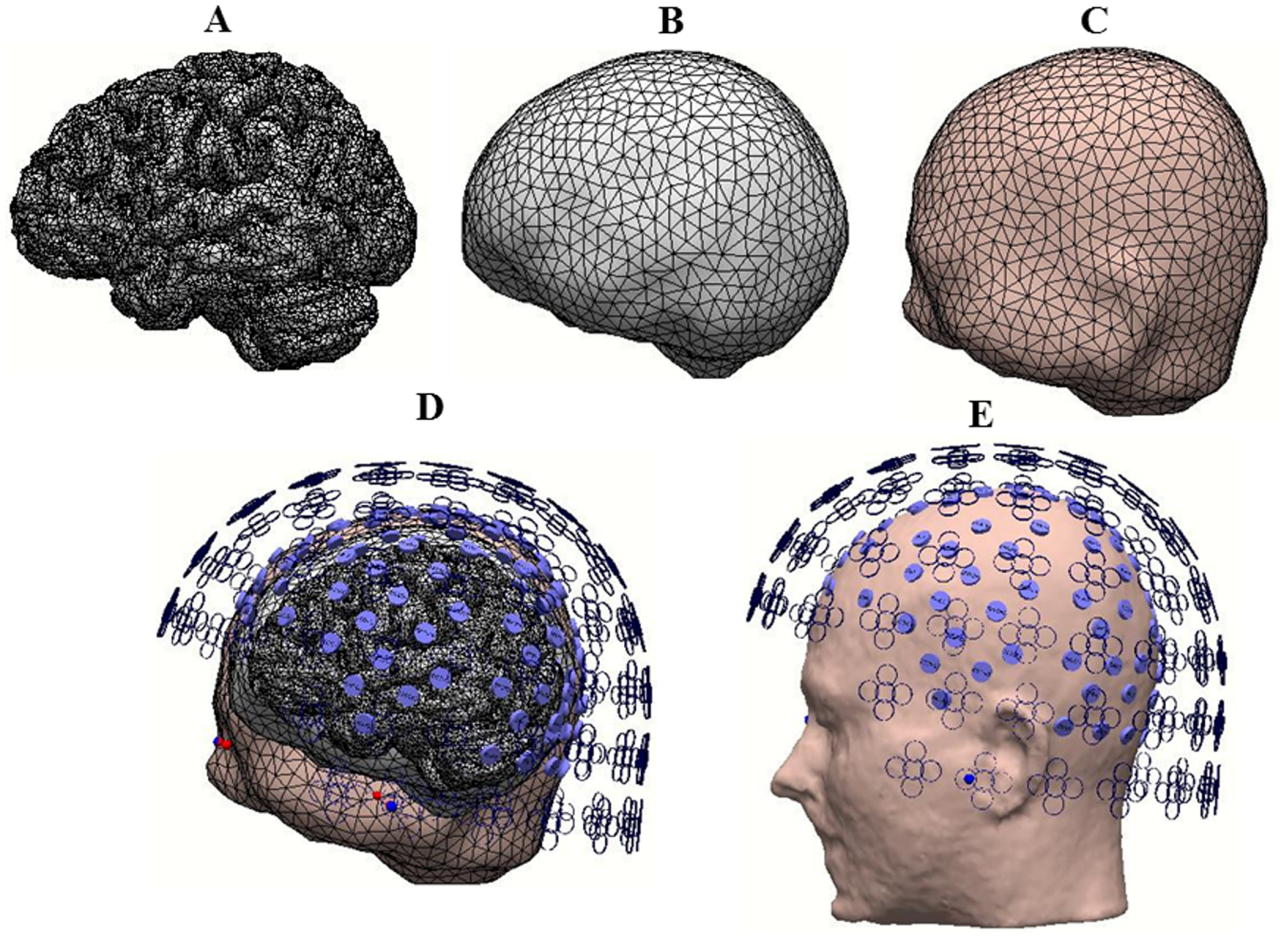
For a representative single subject, the created realistic head model is shown. The layers are represented separately the brain (A), the skull (B), the scalp (C) followed by all the layers (D) with the interpolated electrodes and sensors on the scalp with transparent sections. (E) shows the location of the electrodes and sensors with respect to the subject’s head.

The approach used here is the piece-wise homogeneous approximation which can be solved by using the boundary element method (BEM) [8], [17], [33]–[36]. In the BEM model the conductivity is assumed to be isotropic for each compartment of the head. The lead field matrix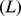 estimated here contains the information about the six parameters (source locations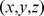, orientations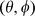, and amplitudes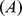) that specify a dipole which models the current sources that can generate the electric or magnetic field pattern at the surface of the head. The surfaces of the compartments like the scalp, skull and brain were extracted from the individual magnetic resonance images (MRI) of each subject. The individual electrode locations for the MEG sensors were recorded automatically from the Neuromag system and the EEG sensor positions were measured by a Polhemus system. The realistic head models were constructed based on the linear-collocation 3-layer BEM model. The main idea of this approach is developed on the basis of the integrated analysis of MEG and EEG simultaneously. The MEG in which the conductivity is a minor concern [17] is used first to find the accurate source location information for the tangential components. Subsequently, this is integrated to obtain the radial component from the EEG data by adjusting the conductivity profile of the EEG model [8]. The conductivity values for the scalp ( = brain) varied from 0.12 to 0.98 S/m and for the skull varied from 0.004 to 0.0013 S/m. The open source software OpenMEEG [37] was used to build the realistic head models. The constructed realistic head model is shown for a representative subject in Figure1. The layers are presented separately the brain (A), the skull (B), the scalp (C) followed by all the layers (D) with the interpolated electrodes and sensors on the scalp. (E) shows the location of the electrodes and sensors with respect to the subject’s head.

### 2.4 Source Analysis

The analysis tool used here is the dynamic imaging of coherent sources (DICS) [19] for identifying the coherent brain sources at the pre-defined frequency band. DICS uses a spatial filter algorithm [38] and estimates the tomographic coherence maps which are based on the realistic head models. There are two major constraints in this beamformer approach: it assumes an un-constrained single dipole model, which is not linearly correlated to other dipoles. This assumption is valid if the coherence is not too strong and the signal-to-noise ratio is sufficient [19]. The second constraint is that the coherence between the identified areas with itself is always 1. The source in the brain with strongest coherence to the EMG signal at the finger tapping frequency (2–4 Hz) was identified. In the next step, this area of the brain or the activated voxels were considered as noise in order to find further weaker coherent areas in the brain [39]. All the coherent brain areas were identified one by one by only taking the EMG as the reference signal, finally their activity was extracted by the spatial filter [38]. The spatial filter was applied to a large number of voxels covering the entire brain, assigning to each voxel a specific value of coherence to the given reference signal (i.e., EMG). A voxel size of 5 mm was used in this study. The dipole orientations for each of these sources were obtained from the resulting lead field matrix for each modality separately and also for the combined approach for each subject. The application of the spatial filter has been described elsewhere [40]. The criteria used to identify areas in the brain was by using the significance level obtained from a within subject surrogate analysis. Local maxima in the resulting maps represent areas that have the strongest coherence to the reference signal. In a further analysis, all the original source signals from each source with several activated voxels were combined by estimating the second order spectra and employing a weighting scheme depending on the analyzed frequency range to form a pooled source signal estimate for every source as previously described in [41], [42]. This analysis was performed for each subject separately, followed by a grand average across all subjects for all the three modalities EEG, MEG and the combined approach (MEG+EEG). All the steps performed in the source analysis are depicted in the flowchart with output pictorial representation after each step in the figure S1.

### 2.5 Renormalized Partial Directed Coherence

To identify the direction of information flow between two signals, the technique called the renormalized partial directed coherence (RPDC) was applied [25]. The multivariate model is strictly based on the principle of Granger causality [43] (i.e., not taking into account zero-lagged or instantaneous influences). The RPDC is a general method mostly used to analyze connectivity of EEG and MEG signals in the frequency domain. The pooled source signals were modelled using an autoregressive process to obtain the coefficients of the signals in the particular frequency band with a multivariate approach. The formulation to estimate the RPDC values between two signals 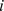 and 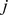 at a specific frequency 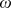 is given as follows [25]:
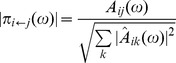
(1)


In the above equation the numerator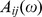 is the estimated autoregressive coefficient from 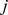 to 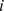 at a certain frequency band . The denominator 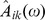 also gives the autoregressive coefficients at the same frequency band 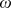 from 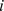 to all other 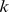 signals, that is, RPDC ranks the interaction strength with respect to the given target signal.

The optimal model order needs to be chosen which was estimated by minimizing the Akaike Information Criterion (AIC). This order indicates the ideal number of coefficients that need to be estimated [44], [45]. The AIC is a measure of the relative goodness of fit which has the minimum loss of information for a resulting statistical model with an optimal order [45]. The bootstrapping method [46] was used to calculate the significance level on the applied data after the estimation of the RPDC values. In the bootstrapping method [46], [47] we divided the original time series into smaller non-overlapping segments of equal size. The smaller windows are shuffled randomly and then concatenated. This process was repeated 100 times and the 99^th^ percentile was taken as the threshold or significance value. The concatenated time series has the same power spectrum as that of the original time series, however all the coherence and directionality is lost. In addition, the significant connections were tested with a time reversal technique only for the EEG modality. In order to justify that the shown connections were due to the strong symmetry present in the data and not due to any volume conduction effects [48]. The open source Matlab (The MatWorks Inc., Natick, MA, USA) package ARFIT [49], [50] was used for estimating the autoregressive coefficients from the spatially filtered source signals.

### 2.6 Signal-to-noise Ratio Analysis

The scalp level relative SNR was estimated for both the modalities separately from the power spectrum of each of the electrodes/sensors. The signal is defined by the peak finger tapping frequency (2–4 Hz) in this task. In order to use a session-specific noise [14] then the noise was estimated from another recording where the same subjects open their eyes without performing any task. The noise level is then defined as the power in the frequency band (2–4 Hz). The same number of sensors was selected from MEG and corresponding EEG electrodes to have a direct comparison of the SNR values. In total 15 electrodes/sensors were selected for the finger tapping task from the contralateral motor cortex region in the scalp. At the end, the mean SNR was estimated from the 15 electrodes/sensors for both the modalities separately. The selection of the electrodes and sensors was done by estimating the Euclidean distance between the EEG electrodes and the corresponding MEG sensors (selected by visualization in the forward model). A sphere with the radius of 40 mm was considered with the center being the EEG-C3 electrode on the scalp. In this analysis, 15 electrodes were selected surrounding the C3 electrode. The criterion was to meet the Euclidean distance< = 20 mm between the EEG channels and corresponding MEG sensors. The source level SNR was estimated by taking the pooled source signals from the identified sources in each modality separately, instead of the electrodes/sensors signals. In case of the combined approach, the SNR was calculated by normalization of the pooled source/scalp signals to their individual noise amplitudes, yielding unit-free measures for both EEG and MEG [10]. The individual noise amplitudes were estimated from the eyes open recording for each individual subject at the frequency band (2–4 Hz).

### 2.7 Time Frequency Analysis

This analysis was performed to find the time segments with higher significant coherence between the EEG/MEG electrodes and the EMG. The dynamics of the cortico-muscular coherence was estimated by the multitaper method [51]. In this method the signals are multiplied initially with different windows (i.e., tapers) (K = 7). The length of the window used in this analysis is 1000 ms. The time step used was 50 ms with overlapping windows of 950 ms, a coherence value is calculated every 50 ms and the frequency resolution is approximately 1 Hz. A 95% overlapping corresponds to a time resolution of approximately 50 ms. The complete description of this method is explained elsewhere [52]. In the subsequent analysis, all the coherence estimates of the significantly coherent EEG/MEG electrodes (selected 15 electrodes/sensors) with the EMG were combined to get a pooled coherence estimate as described earlier in the source analysis section. From the pooled estimate, the time segments (FT-mean: 100±2.4;) were chosen with coherence values greater than (mean+std) for the whole recorded data length. Source analysis was repeated on these time segments for the case of EEG modality only to see whether the analysis identifies sub-cortical sources.

### 2.8 Statistical Analysis

The total data length between the subjects was tested with a non-parametric Friedman test for dependent samples (n = 15, α = 0.01). The significance of the sources were tested by a within subject surrogate analysis. The surrogates were estimated by a Monte Carlo random permutation, i.e., 100 times shuffling of one second segments within each subject. The p-value was estimated for each of these 100 random permutations and the 99^th^ percentile value of each source for all these permutations is taken as the final threshold.

Next, the voxel co-ordinates of the identified sources with the maximum coherence were compared to that of the reference voxel within the same modality. A reference voxel for each of the identified sources was determined in the MNI co-ordinate system for the finger tapping task; [primary sensory motor cortex - PSMC: (−56.0, −14.0, 41.0); premotor cortex – PMC: (−27.0, 46.0, 32.0); supplementary motor area – SMA: (−14.0, −4.0, 44.0); posterior parietal cortex – PPC: (−46.0, −71.0, 35.0); thalamus – TH: (−5.0, −16.0, 8.0); cerebellum – CER: (13.0, −76.0, −51.0)]. The euclidean distance was estimated between the reference voxel and the maximum coherent voxel. In the further analysis, the euclidean distance was estimated between the different modalities, for each of the sources, to compare the difference in the source location (e.g. EEG vs. MEG; EEG vs. EEG+MEG; MEG vs. EEG+MEG). The chi-square variance test was used by defining the category with the minimum distance as zero and the maximum depending on the calculated distance in each combination. Bonferroni correction was done. For three comparisons the level of significance would drop from 0.05 to 0.017 between all three combinations (EEG vs. MEG; EEG vs. EEG+MEG and MEG vs. EEG+MEG).

Finally, the source coherence values and the source signal SNR values (n = 15, α = 0.01) for each of the modalities were tested for significance using the multifactorial analysis of variance (ANOVA), within-subject factors being the sources (n = 4 sources: EEG), (n = 6 sources: MEG and EEG+MEG) and the between subject factor being the modalities (n = 3: EEG, MEG, EEG+MEG). The scalp level SNR between the modalities (EEG vs. MEG) was tested using a non-parametric Friedman test for dependent samples (n = 15, α = 0.01). The mean SNR values of the selected 15 electrodes were compared with the SNR values of the pooled coherence estimate of the time segments with the highest coherence value. These values were tested with a non-parametric Friedman test for dependent samples (n = 15, α = 0.01). The RPDC values (n = 15, α = 0.01) between the pooled source signals were tested for significance using the multifactorial ANOVA, within-subject factors being the connections of the pooled source signals (n = 12 connections: EEG), (n = 30 connections: MEG and EEG+MEG) and the between subject factor being the modalities (n = 3: EEG, MEG, EEG+MEG). The Bonferroni correction was performed for all the post-hoc test which involved multiple comparisons.

## Results

### 3.1 EEG/MEG-EMG Coherence

The data length within the subjects was not significantly different (p = 0.423). Power spectral analysis on the EMG activity of all the subjects showed a dominant peak at the frequency range (2–4 Hz; mean: 2.93±0.70). The cortico-muscular coherence did not differ either in the frequency or in amplitude for both EMG signals with or without rectification. At the above mentioned frequency, all subjects exhibited significant coherence between EMG and EEG/MEG electrodes or sensors covering the region of the contralateral sensorimotor cortex.

### 3.2 Network of Sources

**Figure 2:**
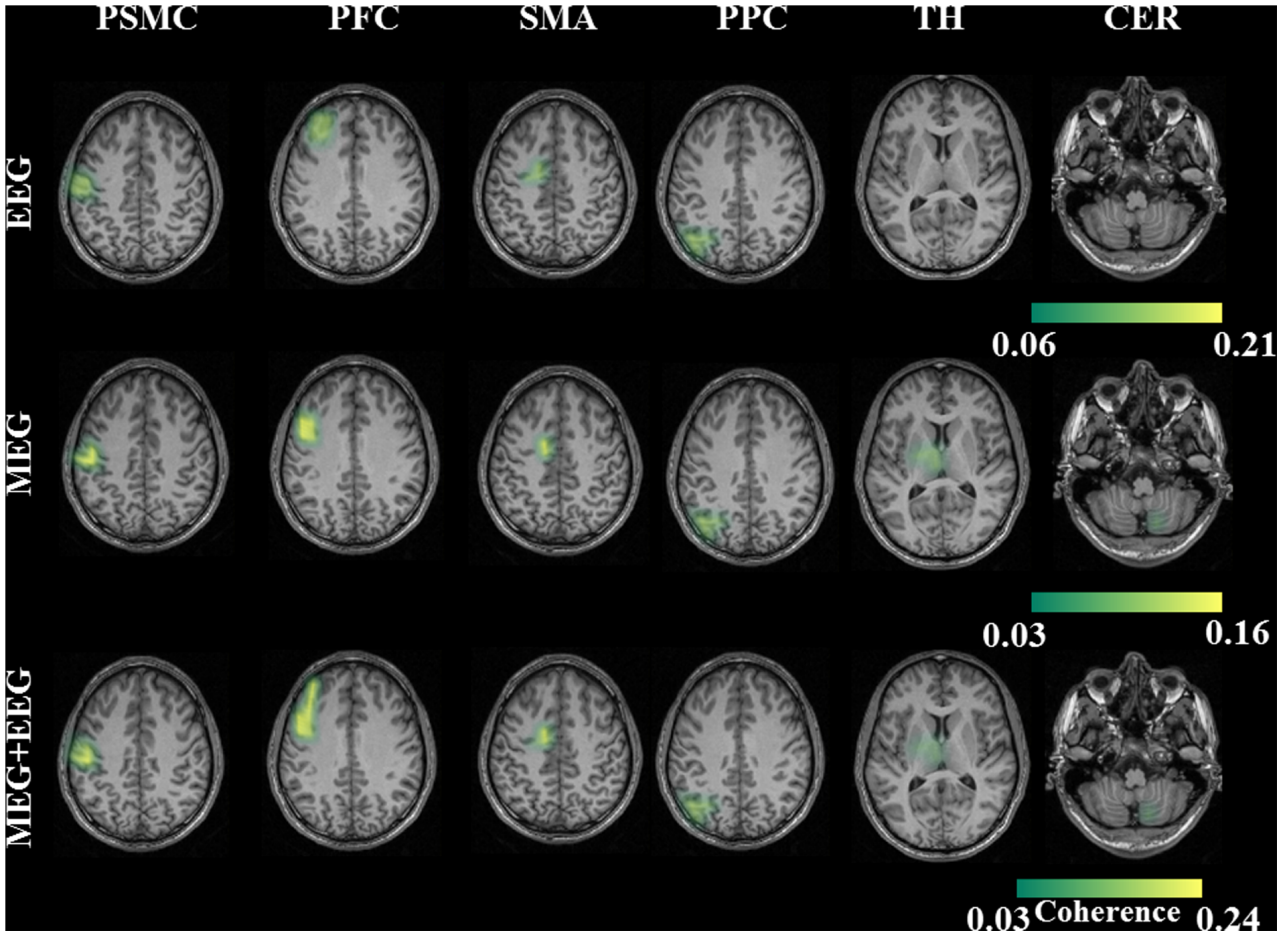
EEG - The grand average statistical map of network of sources for the finger tapping (FT) task by taking EMG as the reference signal for the whole recording data length. MEG - The network of sources for MEG separately. MEG+EEG - The coherent network of sources for the combined approach (MEG+EEG). The network of sources was primary sensory motor cortex (PSMC), premotor cortex (PMC), supplementary motor area (SMA) and posterior parietal cortex (PPC) for the modality EEG. Additionally, two sources at the thalamus (TH), and cerebellum (CER) were identified only for the modalities MEG and for the combined approach (MEG+EEG).

**Figure 3:**
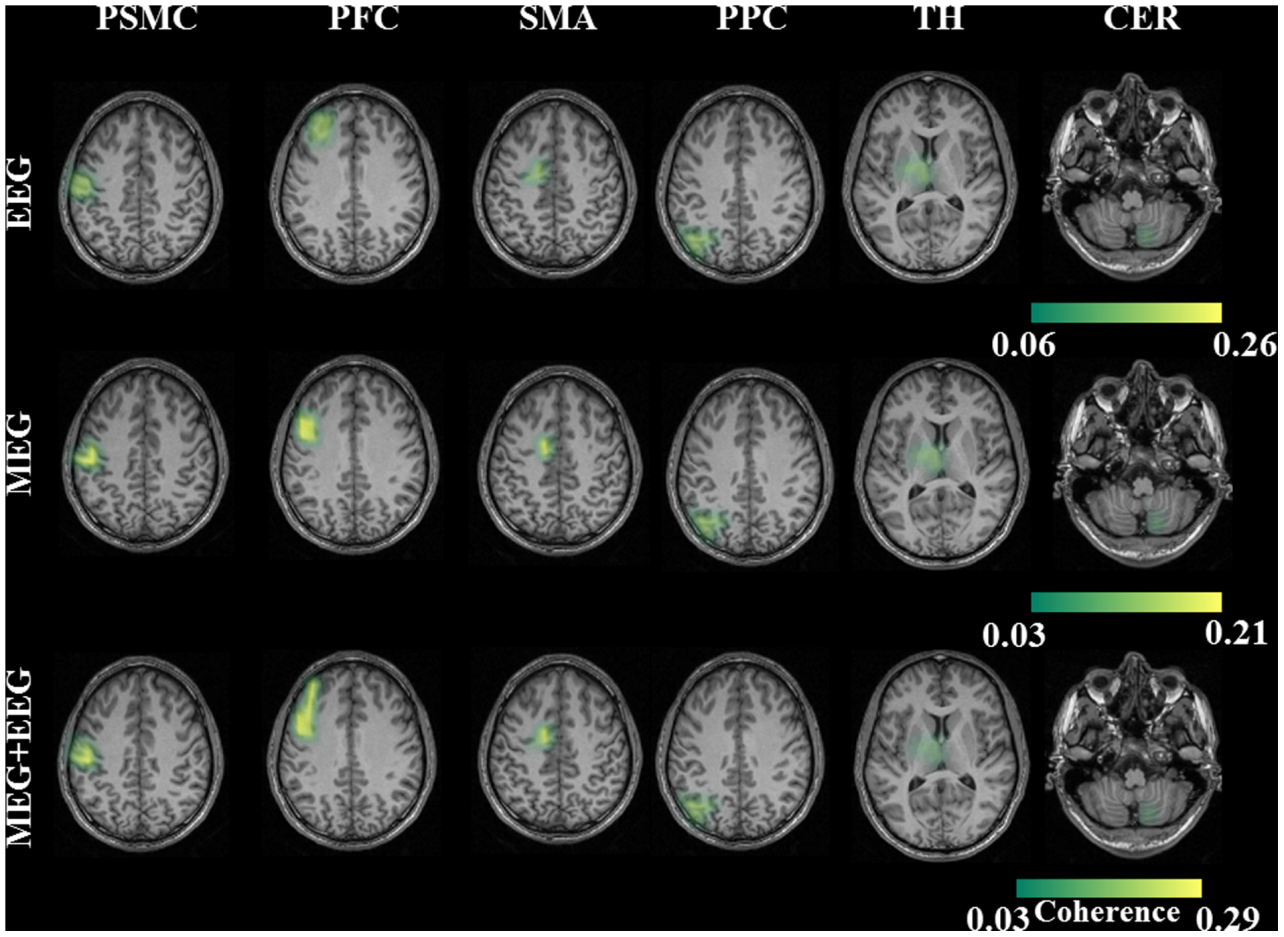
The grand average sources for the higher SNR time segment analysis for EEG, MEG and for the combined approach (MEG+EEG). In this analysis all the modalities showed a similar network of sources.

In all the healthy subjects the network of sources were identified for each of these modalities first separately and then combined. For the EEG modality, the network for the finger tapping frequency consisted of the PSMC (primary sensory motor cortex) Brodmann area (BA) 3, PFC (prefrontal/premotor cortex) BA 6, proper-SMA (supplementary motor area) BA 6 and the PPC (posterior parietal cortex) BA 7 as shown in figure 2. The network for the MEG modality included the first four cortical sources seen in EEG and additional two sub-cortical sources; the thalamus (TH) BA 23 and the cerebellum (CER) in the posterior lobe (right lobule V) (Figure 2). The network for the combined (MEG+EEG) modality also consisted of similar network as that of the MEG modality. This is illustrated in figure 2 group statistics maps of the healthy subjects. All of these identified sources were statistically significant (p = 0.003) in a Monte Carlo random permutation test across all subjects within each modality. For the between subjects same modality test, the euclidean distance of the sources with the reference source was not statistically different for all the sources in all the three modalities; PSMC (EEG-p = 0.76; MEG-p = 0.35; MEG+EEG-p = 0.42); PMC (EEG-p = 0.65; MEG-p = 0.74; MEG+EEG-p = 0.21); SMA (EEG-p = 0.56; MEG-p = 0.63; MEG+EEG-p = 0.4); PPC (EEG-p = 0.49; MEG-p = 0.57; MEG+EEG-p = 0.62); TH (MEG-p = 0.28; MEG+EEG-p = 0.19); CER (MEG-p = 0.35; MEG+EEG-p = 0.45). Thus, this test indicated that the location of the identified sources was not significantly different between the subjects. In a further step, we tested the Euclidean distance for within subject’s using different modalities. All the comparisons between the modalities showed no significant difference; EEG vs. MEG (p = 0.47); EEG vs. MEG+EEG (p = 0.42); MEG vs. MEG+EEG (p = 0.52). This in turn indicated that the different modalities located the sources at the same location either when used separately or combined. The source coherence values for all the cortical and sub-cortical sources for the combined (MEG+EEG) approach had significantly higher (p = 0.009) coherence values as compared to the other two modalities. In the comparison between EEG and MEG, the EEG had significantly higher coherence values for the identified four cortical sources (p = 0.007). The source coherence values for all the sources and the three different modalities are shown in Table 1. The dipole orientation for the identified cortical sources showed preferentially radial sources (60°–120°) for the EEG modality and mostly tangential (1°–60° or 120°–180°) for the MEG modality as expected. In the combined (MEG+EEG) approach the cortical sources predominantly showed tangential orientation (range-n = 8–10; mean: 9.4±1.5 subjects) than radial orientation (range-n = 3–5; mean: 4.12±0.9 subjects). However, in the sub-cortical sources both for the MEG alone and the combined approach all the subjects showed tangential orientation for both TH and CER. The additional selected higher SNR time segment source analysis which is described in (section 2.7– Time frequency analysis), for the case of the EEG alone revealed two further sources in the thalamus (TH) and the cerebellum (CER) for all the subjects as can be seen in figure 3. Comparison between EEG and MEG for the sub-cortical sources, the MEG had significantly higher coherence values for the identified two sub-cortical sources (p = 0.009). The source coherence values indicated that the combined approach produced the optimum results as compared to either of the modalities separately in this specific voluntary task.

**Table 1:** The source (mean±std) of the coherence values for each of the sources and the three different modalities are depicted.

Sources	Modalities		
	EEG	MEG	MEG+EEG
PSMC	0.18±0.04	0.15±0.03	0.22±0.04
PMC	0.16±0.03	0.13±0.04	0.19±0.03
SMA	0.11±0.02	0.10±0.02	0.16±0.03
PPC	0.09±0.02	0.09±0.02	0.13±0.04
TH	–	0.07±0.01	0.10±0.03
CER	–	0.04±0.01	0.08±0.04

The source analysis was repeated by taking only 102 EEG channels and separately 102 gradiometers or 102 magnetometers into consideration. The selection was done on the basis of matching the EEG channels that overlay the MEG sensors according to the measured realistic individual 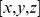 co-ordinates with the Euclidean distance <20 mm. The network of sources identified by both EEG and MEG (magnetometers or gradiometers) were similar but the spatial resolution (no. of voxels included in a single source) was reduced (i.e. more voxels for one source) compared to the analysis with all the electrodes and sensors. The p-values are given first for each of the comparisons followed by the information in square brackets which sensor configurations were compared for the source analyses. For all the sources in the case of EEG there was no significant difference (p = 0.21) [selected 102 EEG channels with all 128 EEG channels], however, in the case of the MEG magnetometers (p = 0.004) [102 magnetometer sensors with all 306 MEG sensors], gradiometer1 (p = 0.006) [102 gradiometer1 sensors with 102 gradiometer2 sensors], gradiometer2 (p = 0.009) [102 gradiometer2 sensors with 102 gradiometer1 sensors] MEG (magnetometers)+EEG (p = 0.008) [102 magnetometer sensors+selected 102 EEG channels with all 306 MEG sensors and 128 EEG channels], MEG (gradiometer1)+EEG (p = 0.009) [102 gradiometer1 sensors+selected 102 EEG channels with all 306 MEG sensors and 128 EEG channels], MEG (gradiometer2)+EEG (p = 0.007) [102 gradiometer1 sensors+selected 102 EEG channels with all 306 MEG sensors and 128 EEG channels] there were significant difference in the identified voxels.

### 3.3. Signal-to-Noise Ratio

The scalp level SNR from the selected 15 electrodes/sensors showed significant difference (p = 0.005; MEG>EEG) between the EEG and MEG for this task. As defined the SNR from the higher coherence time segments showed significantly higher SNR (FT – EEG-0.006; MEG-0.008) than the scalp level SNR for the whole recorded data length using the selected 15 electrodes/sensors. For the source level SNR, the within subject factor, i.e., the first four cortical sources in MEG (namely the CMC; PMC; SMA; PPC) had significantly higher (p = 0.004) SNR as compared to the EEG and the combined (MEG+EEG) approach. The last two sub-cortical sources in the MEG also showed significantly higher SNR (p = 0.007) as compared to the combined approach. The between subject factor, i.e., the modalities, showed that MEG has the highest SNR as compared to the EEG and the combined approach (p = 0.006). The combined approach (MEG+EEG) indicated significantly higher SNR (p = 0.004) than the EEG modality.

### 3.4 Information Flow between Source Signals

**Figure 4:**
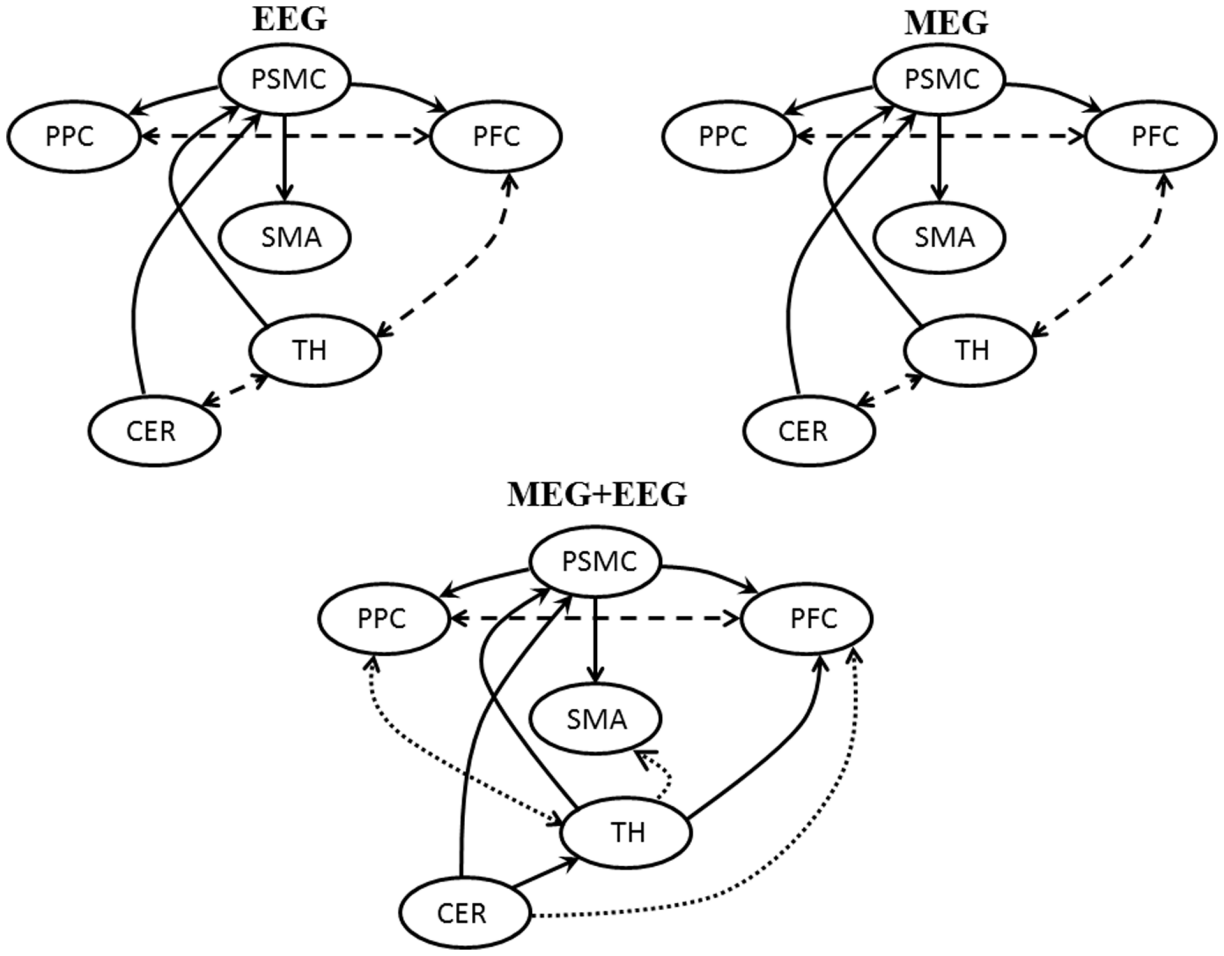
This figure illustrates the information flow between the coherent sources in the brain for the FT task using EEG, Meg and MEG+EEG. The dashed line indicates significant bi-directional interaction and the bold lines with the arrow heads indicate the corresponding significant uni-directional interaction between the sources. The two dotted lines indicate the two additional interactions found between the sources for only the combined approach (MEG+EEG).

The network connectivity between the source signals was estimated using the renormalized partial directed coherence (RPDC) at the desired frequency for the FT task separately for each modality and then combined. The values were statistically compared using ANOVA test for repeated measurements and post-hoc comparisons. For the EEG modality (as shown in Figure 4), the interactions CMC to PMC; CMC to SMA; CMC to PPC were significantly higher for all the subjects (p = 0.004; p = 0.003; p = 0.009) as compared to the opposite interactions. The interactions between the sources PMC and PPC showed significant bidirectional connectivity. The interactions between the sources PMC to SMA and SMA to PPC did not show any significant information flow in both directions. In the MEG modality (as shown in Figure 4), the interactions between the CMC, PMC, SMA and PPC remained similar as in the case of EEG. After the additional time segment analysis, the sub-cortical sources in EEG and MEG, showed significant (p = 0.008; p = 0.009) unidirectional interactions from TH to CMC and CER to CMC. The interactions between the PMC to TH and TH to CER showed significant bidirectional connectivity. The cortical sources SMA and PPC did not show any significant interactions with the sub-cortical sources of TH and CER. In the combined (MEG+EEG) approach (as shown in Figure 4), in addition to the connections found in each of the modalities, EEG and MEG, some additional cortical sources showed significant interactions. The interaction between the TH and CER showed no more bidirectional connection; instead a significant (p = 0.008) unidirectional flow from CER to TH. The interactions between the CER to PMC and TH to SMA showed significant (p = 0.006; p = 0.008) uni-directional connections. The interactions between the TH and PPC were bidirectional (p = 0.004). In the between subject factor analysis, the combined approach (MEG+EEG) showed significantly (p = 0.005) higher RPDC values for all sources in comparison to either of the modalities alone. For the comparison between EEG and MEG, the cortical sources showed higher RPDC values for the EEG modality but the difference was not significant (p = 0.21).

## Discussion

### 4.1 Central Network of Sources Identified by the Different Modalities

The network of cortical sources estimated using each of the modalities separately or combined indicated a similar network. However, the sub-cortical sources were only identified for the MEG and the combined approach (MEG+EEG). These same network of sources have been previously described in EEG [24], [53], [54], MEG [21]–[23], [55], functional magnetic resonance imaging (fMRI) [56], [57] and in a positron-emission tomography (PET) study [23]. In a combined EEG and fMRI study, similar sources were identified for the voluntary movements [54]. These earlier studies describe motor planning in secondary motor centres followed by task execution using the primary sensorimotor cortex. In our earlier studies with EEG alone [24] and MEG alone [21], [22] we were also able to show the involvement of sub-cortical centres, that is, thalamus and cerebellum. However, these sub-cortical sources could only be resolved by the EEG when segments with especially high SNR were selected and analysed separately [24], whereas the MEG resolved these sub-cortical sources more readily without selecting any time segments [22]. The whole cortical and sub-cortical network involved in the finger tapping task has been previously described in voluntary motor tasks [58], [59]. The involvement of Brodmann areas 3, 6, 7, 23 and the posterior lobe (right lobule V) [60] of the cerebellum have been particularly shown to be involved in voluntary rhythmic tasks [61].

This was confirmed in the present study. The additional information to the previous literature is the setting of simultaneous recordings of these two modalities (EEG and MEG) allowing a direct comparison of the spatial location of the identified sources and their corresponding coherence values. The source location were neither significantly different between the subjects nor between the different modalities. This confirms that both approaches detected the same physiological motor network. This also indicated that for identifying the network of cortical sources, the spatial resolution of EEG is sufficient. However, the coherence values for the combined approach were significantly higher for all the sources as compared to either EEG or MEG. In the comparison between EEG and MEG, cortical sources of EEG showed significantly higher coherence values than MEG; and in the sub-cortical sources MEG showed significantly higher coherence values.

### 4.2 Network of Interactions Indicated by the Different Modalities

The network of interactions during the finger tapping task in healthy subjects has been presented in earlier studies using MEG/EEG [21], [22], [24]. In some fMRI studies Granger causality analysis was used to study the effective connectivity during a motor response task [62] and visuomotor task [63]. In most of these studies either part of the network is involved or one of these modalities, EEG/MEG, is used. But, in this work, both these modalities were analysed separately and directly compared with the combined approach which appends to our previous knowledge about the interpretation of the interactions between the network involved in a finger tapping task. Very similar network interactions were revealed when analysing EEG and MEG separately as in these earlier studies [21], [22], [24]. However, the combined approach depicted a few more connections from the sub-cortical to cortical areas. The feedback connection from the TH to SMA and the CER to PMC has been earlier described as direct connections which are hypothesized as a basis of normal oscillatory phenomena [64], [65]. The oscillations induced in the brain due to a finger tapping task are controlled oscillations. The first feedback connection from the main relay nuclei of the thalamus to the (secondary) motor areas is vital for sustaining the coupling between the motor areas and hand muscle. The second feedback from the cerebellum to the pre-motor cortex is vital for the task execution. The bi-directional connection between TH and PPC has been discussed as the connection which maintains the repetitive stability in the brain for motor tasks [22]. This bi-directional connection is important for constant feedback between these two areas which passes on information about the performance of the task from the sub-cortical to cortical centres. These connections were revealed only due to the combined approach which could indicate that more complete network connections are visible when both modalities are used in tandem.

### 4.3 Effect of Signal-to-noise Ratio and Dipole Orientation on Source Analysis

The role of SNR on source analysis has been examined in MEG and/or EEG [11]–[14] with pure simulations or simulated data from real recordings. In this study, we supplement to the beforehand hypotheses that MEG has better SNR by comparing directly the SNR on the scalp and the source level for the finger tapping task. In this task, the MEG showed significantly higher SNR in the scalp and source level as compared to the EEG and the combined approach, whereas, the SNR of the combined signals was significantly better than the EEG. This difference in SNR was reflected in the source analysis. The deeper sources were identified only in the MEG and the combined approach in the initial analysis. The later analysis, with the higher SNR time segments revealed sub-cortical sources in the EEG confirmed the importance of the SNR. These results indicate that EEG is also capable of identifying sub-cortical sources but only with time segments, which have higher signal-to-noise ratio as compared to the whole recorded data length.

In contrast to the signal-to-noise-ratio, the coherence level for the cortical sources was higher in the EEG than in the MEG analysis. Thus the coherence between the cortical motor area and the peripheral muscles is not linearly related to the SNR but may reflect different cortical signal components being picked up by EEG and MEG. On theoretical grounds it is assumed that MEG mostly detects tangentially oriented dipoles and the EEG mainly the dipoles which have a radial orientation [66]–[69]. In view of the cortical anatomy it is self-evident that the signals from the superficial parts of the cortical gyri, the walls of the sulci and the deep regions of the sulci give rise to different dipole orientations and thus the sensitivity of the EEG and MEG to the activity in these sub-regions of the same cortical area also differ [66], [70], [71]. Comparisons between intra-sulcal and gyral electro-corticograms have shown that at least in the primary sensorimotor cortex intra-sulcal and gyral signals seem to be differentially linked to the peripheral motor action [72]. This may be an explanation for the difference in coherence between cortical MEG and EEG observed in the present study and underlines that even in the cortex, which is well-resolved by both methods, they carry complimentary information.

In case of very deep sub-cortical sources, the MEG is clearly superior to the EEG although the dipole orientation of these deep sources is believed to be more radial than tangential [14]. For the detection of these very weak signals coming from the deep region of the brain, an optimal signal-to-noise ratio seems to be the most important prerequisite. Another possible reason in EEG could be the volume conduction effect, which does not affect the MEG system [73]–[75]. In addition, the repetition of the source analysis with only 102 EEG electrodes and 102 MEG sensors (magnetometers or gradiometers) showed no significant change in the spatial resolution.

In conclusion, the SNR is an important parameter when both modalities, EEG and MEG, are used either separately or in tandem. The theoretical dipole orientation in the EEG and MEG modality does not seem to be a major concern as compared to the SNR. The effective connectivity between the sources in the brain also benefits from measuring both these modalities simultaneously. The combination of both these modalities and the use of all the available electrodes/sensors gives the optimum spatial resolution, and also indicates that the coherence could be a useful parameter for identifying the network of sources involved in voluntary motor tasks.

## Supporting Information

Figure S1 **The figure illustrates the steps involved in the source analysis with a pictorial representation of the output after each step.**
(TIF)
