# Supplementary figures and images for: Beamformer Source Analysis and Connectivity on Concurrent EEG and MEG Data during Voluntary Movements
Source: PLoS One. 2014 Mar 11;9(3):e91441. doi: 10.1371/journal.pone.0091441 (PMC3949988; doi:10.1371/journal.pone.0091441)

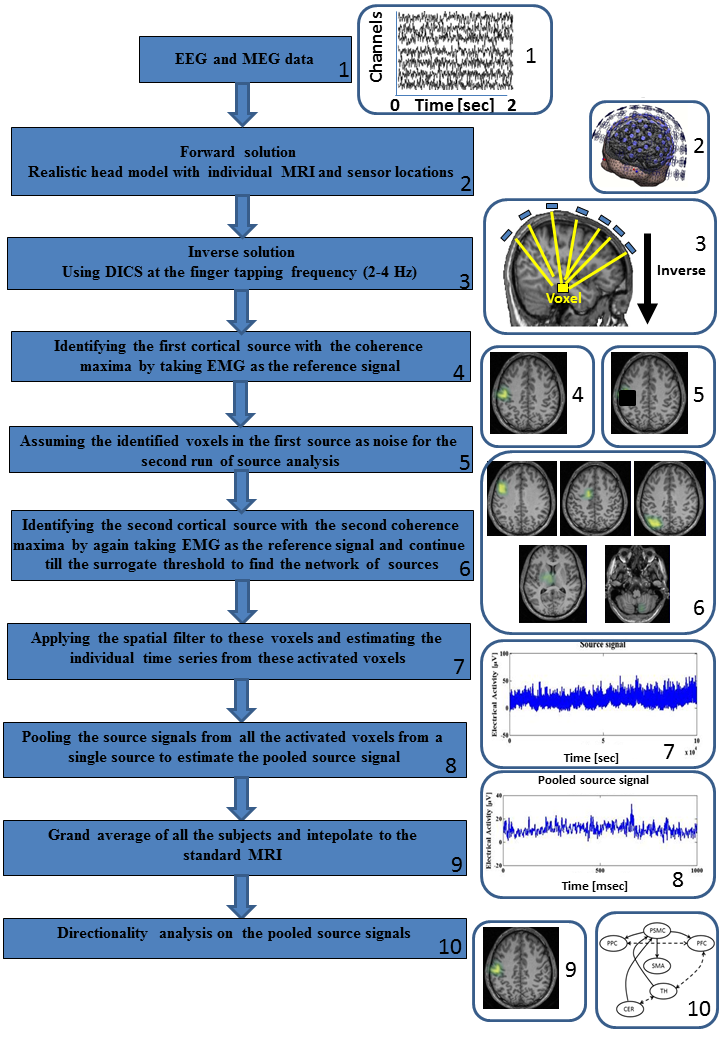

Supplement: Figure S1 — The figure illustrates the steps involved in the source analysis with a pictorial representation of the output after each step. (TIF) [file pone.0091441.s001.tif]
